# Unique Features of a Web-Based Nutrition Website for Childhood Cancer Populations: Descriptive Study

**DOI:** 10.2196/24515

**Published:** 2021-09-13

**Authors:** Lisa Wartenberg, Margaret Raber, Joya Chandra

**Affiliations:** 1 Department of Pediatrics Research The University of Texas MD Anderson Cancer Center Houston, TX United States; 2 Department of Behavioral Science The University of Texas MD Anderson Cancer Center Houston, TX United States; 3 Center for Energy Balance in Cancer Prevention and Survivorship The University of Texas MD Anderson Cancer Center Houston, TX United States

**Keywords:** pediatric oncology, web-based resources, oncology nutrition, culinary education, oncology, children, pediatric, nutrition, culinary, education

## Abstract

**Background:**

Children with cancer experience a myriad of nutritional challenges that impact their nutrition status during treatment and into survivorship. Growing evidence suggests that weight at diagnosis impacts cancer outcomes, but provider guidance on nutrition and diet during treatment varies. Nutrition literacy and culinary resources may help mitigate some common nutritional problems; however, many patients may face barriers to accessing in-person classes. Along with dietitian-led clinical interventions, web-based resources such as the newly updated electronic cookbook (e-cookbook) created by The University of Texas MD Anderson Cancer Center, @TheTable, may facilitate access to nutrition and culinary education during treatment and into survivorship.

**Objective:**

We sought to define and describe the features and content of the @TheTable e-cookbook and compare it with analogous resources for a lay audience of patients with childhood cancer and childhood cancer survivors as well as their families.

**Methods:**

We evaluated freely available web-based resources via a popular online search engine (ie, Google). These searches yielded three web-based resources analogous to @TheTable: the American Institute for Cancer Research’s Healthy Recipes, The Children’s Hospital of San Antonio’s Culinary Health Education for Families Recipe for Life, and Ann Ogden Gaffney and Fred Hutchinson Cancer Research Center’s Cook for Your Life. These sites were analyzed for the following: number of recipes, search functionality, child or family focus, cancer focus, specific dietary guidance, videos or other media, and miscellaneous unique features.

**Results:**

Cook for Your Life and Culinary Health Education for Families Recipe for Life were the most comparable to @TheTable with respect to cancer focus and family focus, respectively. Healthy Recipes is the least user-friendly, with few search options and no didactic videos.

**Conclusions:**

The @TheTable e-cookbook is unique in its offering of child- and family-focused content centered on the cancer and survivorship experience.

## Introduction

An estimated 300,000 children aged 0-19 years are diagnosed with cancer each year in the United States [1]. Although the incidence of cancer in children and adolescents in the United States has risen slightly over the past few decades, the 5-year survival rate has improved from 58% to over 80% since the mid-1970s [2-4]. This significant improvement in survival rate has resulted in the need to mitigate long-term treatment-related sequelae in childhood cancer survivors (CCS), such as secondary cancers, obesity, and cardiovascular disease. Many of these late effects may be exacerbated by excess weight and poor diet, which highlights the importance of nutrition education across the cancer care continuum [5]. Despite these issues, adherence to dietary guidelines is low across the population, including among children without a history of cancer [6], CCS [7,8], and adult survivors of childhood cancer [9-11]. Specifically, the diet of CCS is lacking in fruits, vegetables, and calcium [11,12]. Resources that promote diet quality are scarce for patients with childhood cancer undergoing treatment and CCS as well as their families.

Evidence suggests that children with a BMI outside a healthy weight range at presentation (ie, <5th or >85th percentile [13,14]) have significantly poorer survival than those with a BMI within the healthy weight range [14-17]. Diet quality commonly worsens during treatment in pediatric patients [18] and into survivorship [8,19]; however, provider guidance on diet modification and nutrition adequacy is rarely offered and greatly varies. Recently diagnosed patients and their families have voiced the need to incorporate guidance on appropriate nutrition and lifestyle changes in cancer care [20]. Younger patients may be at particular risk of experiencing adverse effects due to a poor diet that was present and continued through treatment—as many dietary habits are established by the age of 3 years—or one that started during treatment [18,21].

While diet quality in pediatric patients and CCS warrants further research, practical and accessible dietary guidance is clearly needed for patients during treatment. Parents of CCS refer to digital nutrition- and cooking-related content to inform their food choices [22]. Digital information provided by authorized sources could support families of CCS by dispelling diet myths, curbing the spread of misinformation, and promoting nutrition literacy and culinary knowledge. Nutrition-focused web-based resources may help increase the frequency of homemade meals, a factor that has been positively associated with diet quality. Web-based resources focused on healthy eating are promising means to mitigate common treatment-related sequelae and late effects as well as barriers to accessing information on nutrition among CCS.

This paper describes nutrition-focused web-based resources targeted to patients with childhood cancer and CCS as well as their families. The University of Texas MD Anderson Cancer Center’s (MDACC) electronic cookbook (e-cookbook), @TheTable, was developed in 2012 by a team of research dietitians, culinary specialists, and other staff [23]. Since its original dissemination, @TheTable has undergone significant design changes to improve its user-friendliness. The collection of recipes and nutrition-related content has grown substantially, and the educational nutrition information is now offered in three additional languages (Spanish, Mandarin Chinese, Arabic) that reflect well-represented populations within the institution. In this study, we compared the features and content of @TheTable with those of other freely available web-based resources and highlighted the unique aspects of this e-cookbook.

## Methods

### Search Strategy for the Identification of Web-Based Resources 

We mirrored the methods typically used by patients, survivors, and families to acquire information and conducted multiple internet searches between 2019 and 2020 using combinations of keywords such as “tools,” “cancer,” “recipes,” “healthy,” and “eating.” An MDACC research librarian independent of our research team additionally assisted in the search and found comparable results.

### Resource Selection

Resources were included based on the following criteria: a substantial (>100 recipes) and searchable recipe catalogue, cost-free use of resource, and authorized source (ie, content generated or reviewed by a registered dietitian or affiliated with a major institution). From the web-based search, the two closest analogues to @TheTable were selected for in-depth analysis. The included sites were analyzed for the following: number of recipes, search functionality, child or family focus, cancer focus, specific dietary guidance, videos or other media, and miscellaneous unique features. To compensate for the uniqueness of the resources targeted to childhood cancer populations, the Culinary Health Education for Families (CHEF) Recipe for Life site created by The Children’s Hospital of San Antonio was included as a web-based resource with a pediatric focus that the research team was familiar with.

### Features and Content Extraction

Website features are shown in Table 1. Content extracted included number of recipes available on the site and ability to search by certain criteria (eg, texture, symptom, nutrition need, meal type). Filters and search functionality were included as key features of web-based resources, as these allow organization of larger libraries or nutrition content for browsing and also allow users with specific questions or needs to find information quickly, thus increasing usability.

**Table 1:** Key features of web-based cookbooks and resources for patients.

Site title	Author	Recipes, n	Search by symptom	Search by nutrition	Search by meal type	Child focus	Cancer focus	Diet tips/guides	Videos	Other features
@TheTable [24]	The University of Texas MD Anderson Cancer Center	>770	Yes	Yes	Yes	Yes	Yes	Yes	Yes	Question submission
Healthy Recipes [25]	American Institute for Cancer Research	250-300	No	No	Yes	No	Yes	Yes	No	Physical activity guidelines
Culinary Health Education for Families Recipe for Life [26]	The Children’s Hospital of San Antonio	>75	No	Yes	Yes	Yes	No	Some	Yes	Teaching kitchens; provider referrals to culinary programs; home activities; blog; community resources
Cook for Your Life [27]	Ann Ogden Gaffney; Fred Hutchinson Cancer Research Center	>500	No	No	Yes	No	Yes	Yes	Yes	Culturally adapted menus; menu collections

The presence or absence of child or family focus was also noted. A “kid-friendly” website was one that had a sensory approach to food (eg, inclusion of color, shape, texture) to facilitate accommodating child-specific preferences and aversions (related or unrelated to treatment); focused on child-specific nutritional needs related or unrelated to the cancer experience; and acknowledged the role and journey of the caretaker or parent in treatment and survivorship. Cancer focus was gauged according to whether recipes could accommodate treatment-related adverse effects; the presence or absence of symptom- or treatment-specific content or search functions largely defined this category. The availability of nutrition tips or guidelines, the presence of freely available didactic videos, and miscellaneous features unique to the web-based resource (ie, overlapping in-person classes, home activities, blogs or newsletters, cultural inclusivity) were assessed.

## Results

### Included Web-Based Resources

To better examine the MDACC @TheTable e-cookbook, selected web-based resources were analyzed. We identified the following: Healthy Recipes from the American Institute for Cancer Research; CHEF Recipe for Life from the Children’s Hospital of San Antonio; and Cook for Your Life, created by Ann Ogden Gaffney and owned by the Fred Hutchinson Cancer Research Center.

### @TheTable Resource

**Figure 1:**
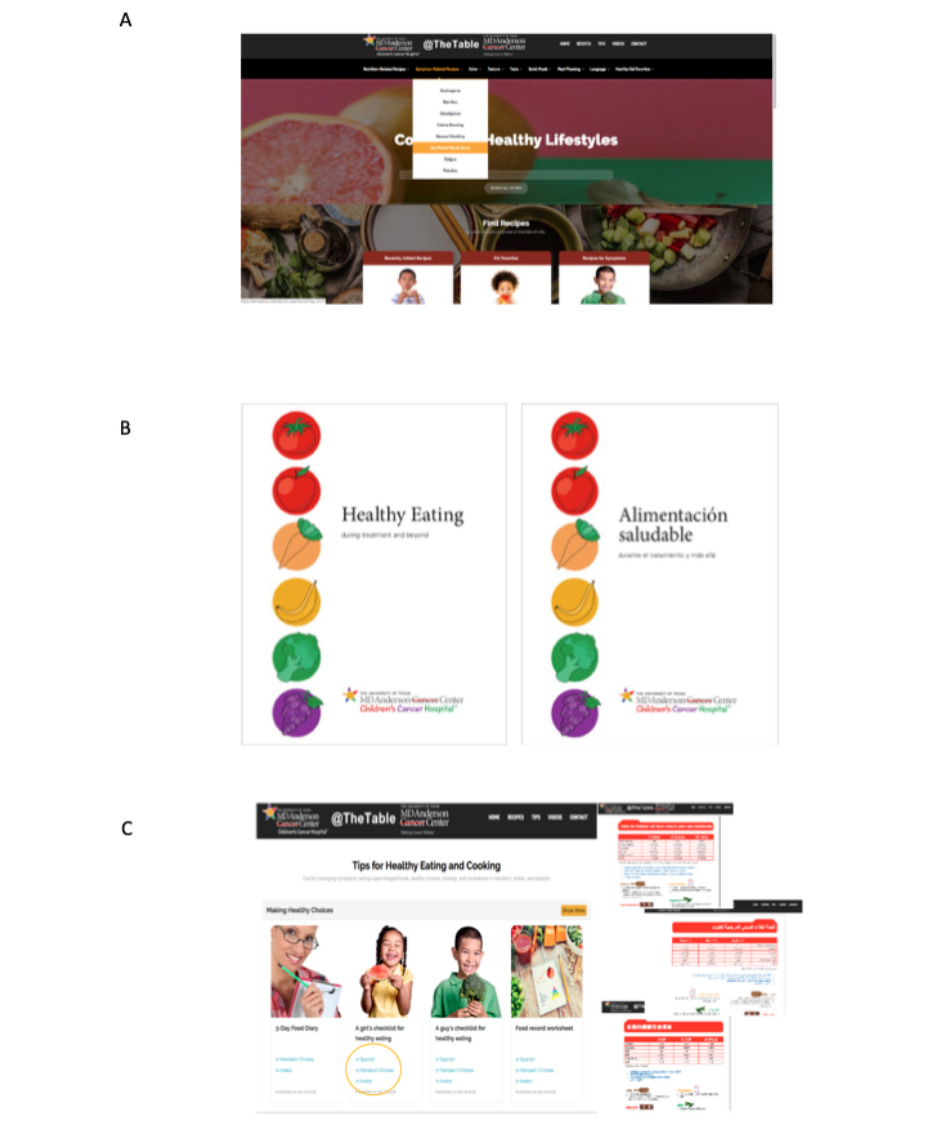
Representative images of @TheTable features for patients with cancer and survivors.

**Figure 2:**
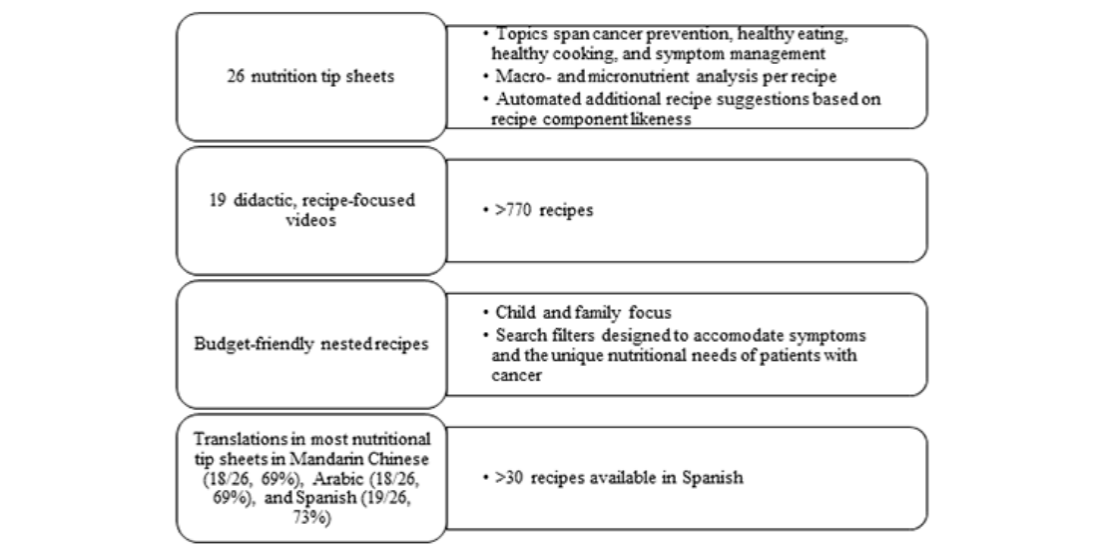
Schematic of additional @TheTable features highlighting its specialty focus and nutritional education, culinary, and multilingual content.

@TheTable is a web-based resource that may be accessed without cost or subscription. It includes over 770 recipes that are searchable by symptom, nutritional need, texture, and flavor, in addition to standard cookbook features (Table 1). Nutrition-related filters allow selection of nutritious recipes specific to certain diets such as vegan, vegetarian, and low-sodium diets (Figure 1). Figure 1A shows a screenshot of the landing page for the @TheTable e-cookbook. From this page, site visitors can access a myriad of freely available resources specifically designed for patients with cancer. The family focus of the e-cookbook aims at bolstering the nutritional habits of family units. The salient features of the e-cookbook are recipes searchable by symptom, meal type, texture, and color, as well as nutrition tip sheets and didactic videos. Most recipes are in English, and some are in Spanish. Figure 1B shows nutrition tip sheets available in this e-cookbook. Freely available tip sheets on @TheTable can additionally be accessed in Spanish, Mandarin Chinese, and Arabic. This augments accessibility, especially for bilingual households or those living outside the United States. Tip sheets cover topics such as the nutritional needs of growing girls and boys undergoing treatment for cancer, the management of symptoms such as constipation, and practical tips for the grocery store. Figure 1C shows the @TheTable nutrition guide for pediatric patients with leukemia. The nutrition booklet follows the pedagogy of MyPlate, the nutrition guide likely used by families in public schools in the United States, and builds on that knowledge. It aims to establish a common language to enhance future interactions with clinicians and dietitians caring for pediatric patients with cancer. This booklet centered around the five food groups is also available in Mandarin Chinese, Arabic, and Spanish. Symptom-related filters show recipes that accommodate common treatment-related adverse effects such as diarrhea, nausea, dysgeusia, and constipation (Figure 2). Site visitors can also use the color filter to search by phytonutrient (eg, blue/purple filter for anthocyanin-rich foods). These specific filters were designed to complement a trifold brochure distributed at weekly in-person MDACC culinary classes. The texture filter allows the user to find comfort measures for symptoms such as mouth sores or mucositis (eg, soft or smooth/creamy) or select sensory aspects (eg, crunchy, dry, chewy). Videos on @TheTable show prerecorded recipes filmed in an onsite research kitchen. These 19 instructional videos feature pediatric patients, chefs or culinary experts, and research dietitians. An additional feature of @TheTable is the nutritional guidance on various topics delivered via tip sheets and booklets (N=26; Figure 2). As Table 2 illustrates, these are also available in Spanish (19/26, 73%), Mandarin Chinese (18/26, 69%), and Arabic (18/26, 69%) to increase usability.

**Table 2:** Breakdown of nutrition education tip sheets available on @TheTable by broad topic.

Health topic	Example(s) of content			Tip sheet quantity	Available translations				
					Spanish		Mandarin Chinese		Arabic
Cancer prevention		Add color to your diet! Fallin’ for pumpkin	4		3	3		3	
Healthy eating		Healthy eating during treatment and beyond booklet What do I eat? A girl’s checklist for healthy eating	10		5	5		5	
Healthy cooking		Meal ideas Prepping vegetables Stock your spice cabinet	10		9	8		8	
Symptom management		Fiber and constipation	2		2	2		2	

### Healthy Recipes Resource

The Healthy Recipes site segments its content into the following two portals that branch off into more focused evidence-based guidelines: cancer prevention and cancer survival. Its recipe search tool is limited to ingredients or meal types; in this way, it does not explicitly focus on the cancer experience. Unlike @TheTable, this web-based resource does not have a child or family focus. Its nutrition-related content is centered around the New American Plate, a broadly used dietary model formulated under the guidance of the American Institute for Cancer Research’s *Third Expert Report*, which translates cancer-specific, evidence-based nutrition recommendations into practical visual aids for the patient or consumer.

### CHEF Recipe for Life Resource

All content on CHEF Recipe for Life is available in either English or Spanish. Recipes can be searched by difficulty level (ie, beginner, intermediate, advanced), meal type, and special diets (eg, dairy-free, low-fat, nut-free). Moreover, its didactic videos offer either recipe instructions or culinary skill tutorials. Users can sign up for newsletters, submit a form to ask a dietitian a specific question that is subsequently addressed in a site blog, or access a provider referral form to enroll in onsite evidence-based classes in the San Antonio area. However, the site focuses on management and prevention of chronic diseases, including type II diabetes and obesity. Its content is thus not cancer-specific, though this site had the strongest community focus of the included sites.

### Cook for Your Life Resource

The web-based resource Cook for Your Life has a substantial recipe collection (>500 recipes) that can be searched by meal type, food preferences, or health considerations. Further, a “menus and collections” feature clusters recipes around commonalities such as “Vegan Kitchen Basics” and “Eat What You Can to Feel Better.” This website stands out for its substantial video collection, which includes skill-building, culinary technique, and recipe demonstrations. Its blog component delivers educational content related to specific food items or ingredients (eg, farro, avocado, curry powder) as well as nutrition guidelines (eg, adequate hydration, survivorship).

### Comparison of Included Sources

All of the discussed web-based resources encourage plant-forward eating. Only @TheTable and Cook for Your Life include the option to filter recipes according to treatment-related symptoms. Like CHEF Recipe for Life, Cook for Your Life has all its content in either English or Spanish, whereas @TheTable has only selected content in other languages. Cook for Your Life and @TheTable both focus on cancer, though Cook for Your Life content is not based on the family or pediatric patient. CHEF Recipe for Life and @TheTable stand out among the included web-based resources for their focus on the family and pediatric patient. Notably, family-centered approaches to prevent obesity have proven to be most effective in pediatric nutritional interventions [28,29]. While @TheTable has cancer-specific content, CHEF Recipe for Life centers on chronic disease prevention and management. Further, the Healthy Kid Favorites search filter found in @TheTable, for instance, allows the user to search for recipes commonly enjoyed by children (eg, macaroni and cheese, pizza, nachos), which are altered to some extent to augment the nutritional profile. For example, the “Chicken Rainbow Nachos” recipe has fiber-packed vegetables with protein-rich chicken and black beans as nutritionally dense additions to the traditional ingredients. Moreover, the recipe is designed as part of a set of “nested recipes,” which begin with a foundational “parent” recipe (eg, Tomato Poached Chicken Filling) that is used to create “child” recipes (eg, Chicken Rainbow Nachos). This approach stems from a community-based intervention with low-income Hispanic families. It allows for economizing and adds variety in home-prepared meals, though it may increase time spent in the kitchen. In addition, @TheTable has over 30 of approximately 770 recipes (~3.8% of the recipe collection) in Spanish, which allows broader user reach.

An additional aspect unique to @TheTable is the ability to search for recipes according to a color associated with a corresponding phytonutrient. This feature can be used independently, though it is meant to complement a physical trifold brochure given to patients and families at MDACC nutrition-focused events such as the onsite weekly cooking classes held in the inpatient unit at the Ronald McDonald House Kitchen. Users can search for orange or red foods, for example, which they learn are rich in carotenoids by reading the brochure. This offers the dual benefit of considering child food preferences as children may be particular about the color of a food or dish. Recipes from this e-cookbook are routinely used in MDACC onsite cooking classes delivered to patients with childhood cancer and CCS as well as their families [30,31].

## Discussion

This paper describes the unique features of the redesigned @TheTable e-cookbook. We compared it to three analogous websites and found that it is unique in its focus on cancer in the pediatric context, as well as search functionality and recipe collection. Further, we found that Cook for Your Life and CHEF Recipe for Life are most similar to @TheTable with respect to cancer and family focus, respectively. Healthy Recipes is the least user-friendly because it has fewer search options than the other websites and no didactic videos.

Web-based resources with a nutrition focus are particularly relevant to pediatric oncology because of the poor diet quality commonly observed in both patients [32-36] and survivors [8,37]. Specialized nutrition resources addressing common treatment-related problems and late effects may help promote healthy eating patterns and improve patient and survivor outcomes [8,15,35,37-39]. While @TheTable and other web-based resources may play an important role in augmenting nutrition literacy and nutrition education in patients with cancer and survivors and their families, these do not supplant dietitian-led nutrition care. These resources are mainly descriptive, which limits conclusions regarding how these resources may be most effectively integrated into patient care.

Web-based resources, such as @TheTable, have the potential for broad utilization in both digital and in-person interventions. For example, @TheTable has been used across diverse settings such as in-person cooking classes at summer camps for CCS, the pediatric inpatient unit, childhood cancer awareness events, and several cooking- and nutrition-focused studies [22,30,31,40]. However, patients and families could face barriers to participating in in-person interventions. Beaulieu-Gagnon et al [41] identified common barriers faced by patients with childhood cancer in attending face-to-face nutrition classes. Notably, low attendance (17 participants over 1 year) resulted in the cancelation of 71% of their healthy cooking courses. Similarly, low attendance is a problem at the in-person weekly cooking classes in the pediatric inpatient unit at MDACC; plans to attend are disrupted for even the most engaged and interested dyads because of unforeseen medical appointments or because the child feels unwell.

A disproportionately higher incidence of treatment-related problems is seen in CCS with low-income or multiracial background [42]. Unfortunately, the need for culturally adapted nutrition- and diet-focused content remains largely unaddressed. Although it is merely a start, @TheTable has most of its educational nutrition content translated into three languages (ie, Spanish, Mandarin Chinese, Arabic), which helps broaden access to this information. Cultural adaptation is imperative for meeting the unique needs of culturally diverse demographic groups. Some innovative digital resources tackle the widespread issue of limited patient access to registered dietitian services [43]. Ina, developed by Savor Health, is a virtual dietitian directed by user input and artificial intelligence. This chat bot provides expert on-demand dietary counseling and recipes via SMS text messaging after patient data is provided. The tools described above highlight ways in which digital applications can potentially enhance patient and survivor care and in turn improve outcomes. Further research is needed to understand the impact of these novel technologies and digital resources.

## Abbreviations

CCS: childhood cancer survivor
CHEF: Culinary Health Education for Families
MDACC: The University of Texas MD Anderson Cancer Center
